# Studying food entrainment: Models, methods, and musings

**DOI:** 10.3389/fnut.2022.998331

**Published:** 2022-09-21

**Authors:** Jacqueline R. Trzeciak, Andrew D. Steele

**Affiliations:** Department of Biological Sciences, California State Polytechnic University Pomona, Pomona, CA, United States

**Keywords:** circadian, food anticipatory activity (FAA), feeding, restricted feeding, time restricted eating, time-restricted feeding

## Abstract

The ability to tell time relative to predictable feeding opportunities has a long history of research, going back more than 100 years with behavioral observations of honeybees and rats. Animals that have access to food at a particular time of day exhibit “food anticipatory activity” (FAA), which is a preprandial increase in activity and arousal thought to be driven by food entrained circadian oscillator(s). However, the mechanisms behind adaptation of behavior to timed feeding continue to elude our grasp. Methods used to study circadian entrainment by food vary depending on the model system and the laboratory conducting the experiments. Most studies have relied on rodent model systems due to neuroanatomical tools and genetic tractability, but even among studies of laboratory mice, methods vary considerably. A lack of consistency within the field in experimental design, reporting, and definition of food entrainment, or even FAA, makes it difficult to compare results across studies or even within the same mutant mouse strain, hindering interpretation of replication studies. Here we examine the conditions used to study food as a time cue and make recommendations for study design and reporting.

## Introduction

Given the over-abundance of food in modern society, it is easy to lose sight of the importance of feeding to animals in nature and in sculpting our own evolutionary history. For most animals, food is extremely scarce and being wise to opportunities to eat is essential for survival. As such, the circadian system has evolved to receive a number of different stimuli—i.e., light, temperature, and food—to keep biological processes coordinated and allow for adaptation to ever changing conditions (1, 2). Food is an often neglected zeitgeber, being outshined by light, which is more intensively studied and much better understood (3). The light entrainment pathway works *via* excitement of intrinsically photosensitive retinal ganglion cells that project to the suprachiasmatic nucleus (SCN), which is the major regulator of circadian rhythms (4). The SCN serves as a light entrained oscillator (LEO) and its near 24-h neuronal activity is so robust that it persists in explanted tissue for more than a year (5). Application of the same logic used to tease apart the neural circuitry and molecular clockwork of the LEO has not yielded great success when applied to studies of the food entrained oscillator(s) (FEO) (6). In this review, we describe attempts at delineating the circadian biology of food entrainment using a multitude of model systems, including extensive use of rodents, and given the wide range of methodology we make suggestions for improvements and considerations in methods and interpretation of data.

## Model systems used to study food entrainment

FAA research has a rich history with early published descriptions of the behavior dating back 100 years (7). In this time an array of organisms of varying complexities have been studied. Honey bees (*A. mellifera*) have been shown to entrain to restricted feeding schedules and exhibit phase differences in expression of cryptochrome2 (cry2) and period (per) with time-restricted foraging (8, 9). By contrast, studies in *D. melanogaster* show that restricted food availability is not able to entrain activity cycles (10, 11). Food anticipation has also been well-documented in fish. This behavior was first explored in 1964, when a pre-meal peak in locomotion was observed in both largemouth bass (*M. salmonides*) and bluegill (*L. macrochirus*) (12). Other studies have followed suit, utilizing goldfish (*C. auratus*) (13, 14) and zebrafish (*D. rerio*) (15, 16), among other species, to attempt to tease out an FEO separate from the LEO. Studies have demonstrated that fish align their activity periods with mealtimes; however, the presence of a FEO in fish that is separate and independent from the LEO is still an open question. Some unconventional mammalian systems have also been utilized; a recent study employed a pig model to show that ghrelin antagonist administration lowered FAA (17). While the bulk of research in the field has been conducted on rats and mice, alternative rodent models like Syrian hamsters (*M. auratus*), gerbils (*M. unguiculatus*), and the diurnal rodent *A. ansorgei* have been helpful in demonstrating the widespread nature of FAA and testing variables such as age and photoperiod (18, 19). For example, Syrian hamsters may express lower FAA under restricted feeding conditions due to a stronger sensitivity to the masking effect of daytime light conditions (20–23). Syrian hamsters have also been utilized for SCN ablation experiments to confirm that the SCN is not required for FAA in a rodent model aside from rats or mice (24).

Rabbits (*O. cuniculus*) naturally nurse their young once daily in the early morning hours, providing a natural system for studying FAA (25, 26). The eyes and ears of rabbit pups remain sealed until postnatal day (PD) 10; in addition, while retinal projections to the SCN are present at birth, its response to photic input is not fully developed until PD12 (27). Furthermore, circadian cycling of clock genes *Per1, Bmal1*, and *Cry1* in the SCN lacks a clear rhythm until PD45 (27). Even with the lack of rhythmic clock gene expression, young pups develop increased preprandial locomotor activity, altered expression of hormones such as ghrelin and corticosterone, and induction of c-Fos expression in several hypothalamic nuclei, including oxytocin neurons in the paraventricular nucleus (PVN) (28–31). These factors have made the rabbit pup an important model in which to explore the FEO with minimal interaction from the LEO.

Rats (*R. norvegicus*) have been a vital system for studying food entrainment and have been utilized for lesion experiments that provided many key negative results, ruling out structures of interest. Rats with lesions of the SCN lose their light:dark (LD) entrained rhythms when fed *ad libitum*, but maintain robust FAA when given restricted food access (32–34). These findings were repeated in mice (35), supporting the presence of a FEO separate from the LEO and that the SCN is not necessary for the development of food entrainment. Further ablation experiments have been conducted in rats in an attempt to find a region of control for FAA, showing that ablation of the olfactory bulb, dorsomedial hypothalamus, and thalamic paraventricular nucleus, as well as vagotomy, fail to prevent FAA (36–41). Historically these studies have lacked consistency, both in methodology and results, and as such the existence of a single structure regulating food entrainment remains a theoretical possibility (42).

Due to their increased size and complexity, many experiments have been completed in rats that would likely not be possible with mice. In a recent study, rats were taught to press a lever for food and then subjected to restricted food access, using lever pressing as a measure of FAA; the lever pressing showed a preprandial increase, even in animals with SCN lesions (43). Rats have also been shown to be more attuned to entrainment by palatable meals, developing FAA to the presence of a palatable treat provided in addition to standard chow *ad libitum* (44, 45). Another advantage of rats is their increased size in comparison to mice, making them more suitable for meal withdrawal experiments, which helped to rule out “hourglass” timers for food entrainment (46). Withdrawal of food is a key element to proof of entrainment; however doing so in mice, which are much smaller and extremely metabolically active, causes rapid weight loss and protocols with this withdrawal period are often not approved by local research ethics boards.

Even within the laboratory mouse (*M. musculus*), there is substantial variation in genetic makeup that has been shown to affect any number of phenotypes (47). Most studies of FAA in mice are done on the C57BL/6 background, with many utilizing the J substrain from Jackson labs; however, the BTBR strain, which is a popular model of autism (48), was recently shown to have superior FAA compared to C57BL/6J (49). Moreover, phase shifting experiments also demonstrated that the BTBR strain was better able to adjust to altered light or meal delivery times when compared to C57BL/6J mice (49). BALB/C mice are believed to have weakly-coupled circadian oscillators controlling their light-based rhythms (50). When these mice were used to study FAA, they quickly developed high levels of FAA when compared to the C57BL/6J strain (51). The inbred 129S1 strain has been tested under 60% calorie restriction (CR) conditions and showed that they redistributed almost all high activity behaviors to the pre-meal window, displaying “exceptional” FAA (52). Historically, it has been shown that C57BL/6(J) mice have relatively weak FAA when compared to rats (35), which begs the question- why has this been the primary mouse strain utilized for studying FAA when other strains are available that are more attuned to scheduled feeding? We suggest that researchers continue to explore alternative strain backgrounds and consider using different strain backgrounds that demonstrate stronger FAA. Furthermore, prior research has found both major and minor variations in the genomes of commonly used strains of mice, such as C57BL/6J (B6/J) and C57BL/6N (B6/N). For example, a common mutation in *nicotinamide nucleotide transhydrogenase* (*Nnt*) gene, which is found solely in the B6/J strain, appears to negatively impact mice in metabolic studies (53). Mice with the *Nnt* mutation have an increased chance of diet-induced obesity due to glucose intolerance, as *Nnt* is necessary for the maintenance of ATP synthesis (53, 54). On the other hand, B6/N mice carry a frameshift mutation in *retinal degeneration 8* (*rd8*) and in the *cell polarity complex component* (*Crb1*), impairing vision (55). The effects of these particular mutations have not been studied for their effect on FAA.

Pendergast and Yamazaki (6) provided an authoritative review of food entrainment studies of mouse mutants, summarizing every knockout (KO) mouse tested (6). As a thought experiment, we describe the idealized mouse mutant that we have hoped to discover through genetic approaches to studying food entrainment. Firstly, this mouse would have normal light entrained rhythms and its total activity levels would be unaltered. Secondly, both its feeding, body weight, and body composition would be indistinguishable from controls. Third, its digestive and nutrient absorption system would be fully intact. Finally, it would have a complete deficit in predicting scheduled mealtime at a behavioral level–both when fed in the light cycle, dark cycle, and under constant conditions (DD or LL). Peripheral oscillations, such as liver gene expression, corticosterone secretion, and body temperature would all show normal entrainment. Whatever gene deletion is present in this mouse would be recapitulated in a conditional KO where this gene was removed from only a subset of neurons, indicating a brain region(s) that serves as the FEO. Alas, we are lacking such a mouse, but important strides have been made in this direction, including a mouse mutant lacking *Nr1d1* in neurons that cannot predict scheduled mealtime when fed during the day (56).

## Food entrainment methodology in rodents

Since we study FAA in rodents, we are focusing on this system here but the methodological issues described herein can be applied similarly to other organisms. For studies of food entrainment, experimental conditions vary considerably. For example, temporal restriction (RF) of feeding is the most common method, but the duration of food availability has a large range. Similarly, some studies offer a larger temporal window of food availability that tapers down over time while others do not. Calorie restriction (CR), feeding anywhere between 60 and 80% of normal food intake, is another commonly employed method for inducing FAA. CR has the advantage of fewer handling steps (one feeding vs. delivering and removing food) but the disadvantage is that mice can potentially “ration” their food and may not consume it all during a tight window (we have not observed this to be the case after the first few days of CR). Despite these concerns, a side-by-side comparison of body temperature and FAA in mice on 60% CR vs. 3-h RF showed similar entrainment of activity and body temperature to feeding (57). Multiple feedings can also be employed: for example, Luby and colleagues demonstrated that mice can anticipate several meals per day but not with high precision while Petersen et al. demonstrated that rats are much better at anticipation of multiple mealtimes (43, 58).

Regarding RF, the temporal window of food access and when that occurs during the LD cycle varies a lot from study to study. At one extreme would be studies like that of del Rio-Martin et al. (59) that fed mice during the entire 12 h dark cycle, leading to minimal FAA in controls and the erroneous conclusion that a mutant mouse (*Pitx3*^*ak*^) failed to show metabolic or behavior entrainment to scheduled feeding (59). When we studied the same mutant under a CR feeding regimen for many days (>40 of 60% CR), we observed robust FAA in the mutant and controls, highlighting the importance of feeding protocols and duration of experiments (60). Other studies like those of Li et al. (61) and Kaur et al. (62) give a 4-h access to food, but the overall duration of their experiments are quite short, lasting only 9–10 days of RF (61, 62). Such short duration studies can be problematic. For example, when we studied the same orexin KO mouse used by Kaur et al. (62), it had a slight delay in acquisition of FAA by about 1 week compared to littermate control mice (57). However, by 2 weeks of timed 60% CR, there were no differences between groups; moreover, the orexin KO showed resistance to weight loss on CR, making it difficult to interpret this delay in establishing FAA. Another important point discussed by Pendergast and Yamazaki (6) is the use of fasting days both pre- and post RF. For example, fasting a mouse for 24 h prior to beginning RF reduces both fat and lean mass and leads to much more rapid appearance of FAA, even within a few days (63). Some laboratories even combine temporal and calorie restriction (64). Finally, it will be important to examine the effect of repeated testing on the same animals for parameters of food entrainment. For example, LeSauter and colleagues concluded from their studies of D2 dopamine receptor overexpression transgenic mice that this manipulation lowered “motivation” for food, but in their studies they repeatedly tested the same mice in a short- (4 h), medium (6 h), and long-duration (8 h) RF schedule, finding that only under the longest duration RF that the D2 transgenic mice had less FAA (65). Between rounds of RF, *ad libitum* access to food was given for variable amounts of time, ranging from 7 to 10 days but also including a fasting day in one of these respites (65). Expression of FAA should not necessarily be interpreted as lower motivation and it is important to apply instrumental assays to test this more explicitly.

Does restriction amount or the amount of weight loss matter? In a study using C57BL/6J male mice, we compared 60 vs. 80% CR fed in the middle of the light cycle (66). Both the 60% and 80% CR groups demonstrated FAA within 7 days of scheduled feeding, losing about 10% body weight during that first week and weight loss continued to decline for both groups until day 14. However, the 80% group's body weight rebounded slightly as the study progressed and their level of FAA declined after day 14, whereas weight loss in the 60% group continued and the magnitude of their FAA was almost twice that of mice on 80% CR at later time points when both groups had clearly developed FAA. Thus, the amount of restriction and accompanying weight loss appeared to dictate the amount and maintenance of FAA. However, and quite unexpectedly, examining individual mice within either the 60% or the 80% group showed that there was no correlation between the amount of weight loss experienced by each individual mouse and the magnitude of FAA that it expressed. Thus, weight loss or at least having mice in a negative energy balance appears to be an important aspect of obtaining maximal FAA (i.e., 60% has greater FAA than 80% after day 14), but at an individual level the weight loss is not predictive of the amplitude of FAA. In the future, a study that explicitly tests the effect of starting body weight on food entrainment would be of interest. For example, a cohort of same-aged mice could be either over-fed, under-fed (without a timing component), or normally fed prior to entering a timed feeding study. This would allow for comparison of overweight, normal weight, and under-weight status on induction and maintenance of FAA. It is important to note that some laboratories distinguish between hypo-and normocaloric food entrainment and obtain some measure of food entrainment by scheduled feeding that does not result in a net negative energy balance (67).

Diet plays a large role in metabolism and has effects on feeding behavior. The ability to achieve FAA on specific diets corresponds with a macronutrient restrictive food anticipatory study in which subsequent restriction of protein, carbohydrate, and fat nutrients in rats did not substantially affect locomotor behavior prior to reward stimulus (68). Binge-eating behaviors can lead to obesity and often stem from not only the addictive behavior of eating at a specific time during the day but even just from the sight or smell of palatable food (69). In the past, Mistlberger et al. attempted to observe the potential effects of food in relation to activity in mice by testing rats on two different types of diets. The main difference between these two diets was the lack of a simple sugar in unformulated or standard mouse chow, whereas formulated chow had sucrose. Rats in this study were deprived of either protein, carbohydrates, or high fat diet for about 2 weeks, then introduced to the missing macronutrient and tested for locomotor behavior using tilt cages (68). In other words, a palatable meal offered during caloric restriction is sufficient as an entrainment cue for food anticipation. These findings suggest an independence exists between a single macronutrient and FEO(s), thus diet in itself is not a sufficient cue to entrain any FEO(s).

High-fat palatable meals and “treats” have been shown to be a strong promoter of FAA. Palatable meals are loosely defined as being nutrient-rich with early studies utilizing a “nutrient-rich palatable mash” (45), but have included a variety of formulas such as high-fat rodent diets and treats like chocolate and cheese containing varying levels of sugar and fat content (52, 70). Overall, rats and mice have been shown to develop FAA and binge-type eating with the addition of high-fat meals, with correlating c-Fos activation in reward centers of the brain (44, 52, 71). A recent study also showed that, in rats, a high-fat diet stimulated higher c-Fos expression, stronger FAA, and more binge-type eating than a high-sugar diet (72). That being said, restricted access to a high-sucrose meal is known to activate c-Fos expression in the prefrontal cortex, lateral septum, nucleus accumbens and anterior lateral hypothalamus during the pre-meal window (73). It would be of interest to test whether the palatability, sweetness, and formulation of the diet would influence food entrainment, in particular the amount of FAA.

## Factors other than food

Aside from diet and feeding times, there are a number of other variables that have been shown to impact FAA but are not always reported or considered. Overall, sex differences in FAA in rodents are modest, but should be studied and reported. Mice on RF schedules indicate clear differences between the sexes in regard to food intake, body weight, and food anticipation (61). This experiment, which separated male and female mice, depicts males to be generally more active and to have exhibited greater wheel-running activity during RF (61). Another study focused on sex differences in D1 receptor (D1R) KO mice also showed that female D1R KO mice had more severely attenuated FAA than the D1R males (74). In addition, ghrelin levels, the hormone responsible for triggering an appetite, were analyzed to be higher in female than male mice (61), which suggests gonadal hormonal differences can account for sex-related differences in regards to FAA. Supporting this, gonadectomized mice were observed to have similar food anticipation levels over a span of 10 days of RF (61). However, a series of follow-up experiments opposes this finding that differences in FAA are related to gonadal sex hormone differences. Instead, Aguayo et al. showed that singular manipulations to gonadal hormones, sex chromosomes, and developmental patterning in WT mice are not enough to explain the sex differences (75). This study used gonadectomized mice, sex chromosome copy mutants, and masculinized female mice that were treated with 17-β estradiol during their neonatal period and still reported no differences between males and females while on a calorie-restricted feeding paradigm (75). Therefore, while there are reports that indicate male mice to be generally more active than females, gonadal hormone differences alone cannot account for these findings. We showed that sex difference in FAA was actually age dependent. Female mice that were 9–11 months old no longer had a decreased level of FAA compared to male mice (75).

In addition to the loss of sex differences in mice aged 9–11 months (75), age has also been shown to reduce amplitude and onset of FAA. A study comparing FAA in young and aged rats showed that rats aged 24–25 months, being of extreme old age, had a longer onset time and a lower amplitude of FAA when compared to rats aged 3–21 months (76). Another study examined FAA in dysrhythmic rats aged 13–18 months compared to young control rats and young SCN-lesioned rats; here, they found that the aged rats were able to develop FAA and successfully entrain to a restricted feeding schedule, although aged rats with intact LD cycling were not run in concurrence to compare onset and amplitude of activity (77). As it has been shown that aged mice display a decreased capacity for re-entrainment to LD phase shifts (78) it would stand to reason that the same would be true for entrainment to feeding schedules.

Manipulations in photoperiod have shown that the duration of the light cycle has a minimal effect on FAA and that the behavior is able to persist in both constant conditions (both LL and DD) (79, 80). Mice have also been tested in long- and short-day photoperiods, but neither consistently affected FAA (81). Interestingly, skeleton photoperiods enhanced FAA though an unknown mechanism (82).

Numerous studies of the gut microbiome have shown that time-restricted feeding or addition of specific microbiome derivatives (83) are capable of altering expression patterns of core clock genes both *in vitro* and *in vivo* [reviewed in Dass and de Ross (84)]. It has been shown that mice given standard chow *ad libitum* exhibit cyclical expression of gut microflora and that *ad libitum* access to high fat diet reduces these cyclical fluctuations; however, animals given time-restricted access to the same high fat diet retain the cyclical expression of the microflora (85). Another study demonstrated that disruption of LD rhythms by regular phase shifts resulted in changes in intestinal microflora only when mice were fed a high-fat diet, while mice fed normal chow *ad libitum* did not experience a similar change (86). In this study, it was discussed that a secondary stressor on top of phase shifting, such as alcohol consumption (87), high-fat & high-sugar diet, or general stress is often required to induce these microbiome changes and they may not occur in an “unchallenging” environment (88). While neither paper examined the gut microbiome of mice on a restricted feeding schedule of normal chow, given the stress likely induced by single housing and food restriction regimens, it would follow that such conditions may also induce changes in the gut microbiome of animals undergoing a “typical” FAA experiment, some of which may be cyclical as seen by Zarrinpar et al. Given the known interaction of the microbiome and expression of clock genes in peripheral tissues [reviewed in Dass and de Ross (84)], more investigation of how the microbiome affects behavior and FAA may be warranted. Initial studies could measure FAA in germ-free mice to establish the necessity of gut microbiota for food entrainment.

Other factors to consider are the activity measurement method and cage environment. For example, we observed that higher ambient temperature is suppressive of FAA (66) while others have obtained robust inductions of FAA and other readouts of entrainment at thermoneutral temperatures in mice (29°C) (89). There are a variety of methods utilized for quantification of food entrainment, including but not limited to wheel running, lever pressing, telemetry sensors, photobeams, motion sensors, and video-based homecage behavior analysis (40, 43, 52, 60, 90, 91). Direct comparison between telemetry sensors and motion sensors have shown that quantification through these methods are equivalent (92). Alternatively, the inclusion of a running wheel in a cage has been shown to increase FAA; even mice with previous wheel-running experience showed increased FAA when given a locked running wheel, although mice without prior wheel-running failed to show a similar increase (90). It was also shown that circadian organization, observed by Per2:Luc expression, is affected by wheel running. Phase-shifting of the lung, spleen, and liver was observed and shown to be wheel-running specific, as an enriched environment alone did not produce the same effect (93). A major setback of a number of behavioral analysis systems is the requirement of single housing the mice in a barren cage. One example of this is in Gallardo et al. (66), where video-based homecage behavior is analyzed using the HomeCageScan software (behavioral definitions described in 88). This method allows for a more detailed breakdown of behaviors than simple activity sensors or photobeam breaks, which has shown to be useful in certain mouse mutant lines. For example, in leptin KO mice we observed an increase in preprandial hanging and jumping behavior but walking was not increased (57). The sum of high activity behaviors was not significantly increased in leptin KO mice on CR, because this measurement is dominated by walking behavior. We concluded that the leptin KO mice had an intact FEO because of their timing of jumping and hanging, but other groups have reported that leptin negatively regulates FAA, making their measurements using locomotor activity *via* photobeam breaks (94). In this study leptin KO mice had enhanced FAA, consistent with studies in rats that harbor a homozygous null mutation in the *leptin receptor* gene (95). Administration of leptin blunted FAA in leptin KO mice but leptin administration in wild-type mice had no effect on FAA (94). That being said, there are downsides of video recording behavior that should be mentioned: most video-based methods require an empty cage with minimal bedding, depriving the animals of enrichment (96). In Pendergast et al. (93), three separate measures of activity were taken by way of infrared motion sensors, wheel running, and infrared video analysis of eating behavior. This use of multiple techniques provides several measures of behavior while also allowing for an enriched environment for the animals (93). Given that FAA is likely an adaptation of animals to forage during periods of food availability, providing more forms of enrichment to allow for those behaviors may be ideal. Furthermore, studying food entrainment in socially housed animals has not been conducted to our knowledge, as most measures of activity require individual housing.

## Recommendations and considerations for food entrainment studies

Based on the considerations and methodological issues described above, we advocate for the collection, presentation, and analysis of food entrainment data as follows:

• **Food intake values should be reported as raw and/or normalized to body weight**. These feeding measurements should be reported prior to restriction and during, if appropriate (i.e. during RF it is possible to measure food intake whereas during CR the value is fixed).
• **Although on its own, body weight loss is only loosely predictive of FAA, it is still important to report both as raw data (i.e. grams) and as a percentage of starting weight**. Ideally, measurements of lean and fat mass would be presented as well, especially in cases of mouse mutants that have adipose or metabolic phenotypes (57). For example, it was reported that orexin KO or orexin neuron ablated mice had less FAA (62, 97), but when we tested timed CR in orexin KO mice we observed that they were resistant to weight loss during CR and eventually developed comparable FAA to controls (57). Since starting body weight and/or fat stores can be affected by genetic manipulations and will likely affect the development of FAA, these data need to be considered and reported.
• **With respect to metabolism, measurements of indirect calorimetry (i.e. respiratory exchange ratios) are desirable especially when mutations cause metabolic phenotypes**. We are only aware of a couple of examples of studies of food entrainment that measure energy expenditure (60, 98) and recognize the difficulty in conducting them given the cost of obtaining the equipment. More studies of respiratory exchange ratios and energy usage under different feeding protocols and in different mouse mutants may reveal previously unappreciated principle components of food entrainment.
• **Raw and normalized behavioral activity data should be reported**. This will allow for i) visualizing total activity levels, which are often affected by gene deletions, and ii) for comparison to nocturnal activity peaks as a frame of reference. Thirdly, normalization to total activity corrects for differences within lines of mice and also allows for comparisons between studies (i.e. a 30% redistribution of activity to precede scheduled mealtime). In our study of sex differences in FAA using C57BL/6J mice, we observed no difference in premeal high activity behavior when comparing males vs. females, but when we normalized these data, the females clearly showed less FAA and higher nighttime activity (75).
• As recommended by Pendergast and Yamazaki (6), if permitted by the Institutional Animal Care and Use Committee, **include at least one fasting day or meal omission after FAA has been established** in order to discern if the mutation/manipulation in question is affecting the expression of FAA or the FEO itself.
• **Conduct initial studies of FAA in 12:12 LD conditions with feeding in the middle of the light cycle**. This can be followed by testing FAA for nighttime feeding as some mutant mice have specific deficits in FAA only for light cycle feeding [for example, see Podyma et al. (63)]. Finally, examining FAA in constant darkness or constant light and/or meal shifting experiments are an important third and fourth lines of inquiry that allow for studying FAA with less contribution from the LEO.
• **Conduct longer duration experiments or do a fasting day(s) prior to timed feeding for shorter duration experiments**. Studies of FAA range from less than a week (63) on the low end, to at least 2 months on the high end (60, 64). As starvation increases activity acutely (52), it is important to distinguish whether mice have a phenotype relating to hunger induced activity vs. food entrained activity, thus adding a week of RF/CR to a study can assist with interpretation of results in combination with body weight data (i.e. when weights have plateaued).
• **Report strain information** and track genetic purity of inbred rodent models to monitor genetic background and possible interacting alleles, especially in the context of KO/Cre transgenic mice made from ES cells on different genetic backgrounds where linkage disequilibrium makes it nearly impossible to obtain alleles on a pure background *via* backcrosses (47).
• **Report lab diet formulations**. As discussed above, foods with higher fat content tend to be suppressive of FAA while perplexingly, the palatable treats used to induce FAA in mice need to have high fat content (52, 70). It is also an outstanding question as to whether formulated diets will lead to more consistent or stronger food entrainment.
• **Report sexes and ages of animals**. Females are understudied in circadian biology and metabolism (99). The age of animals affects their size and adiposity, and as discussed above can have an impact on FAA (75).
• **Conduct follow-up and replication studies, both within and between laboratories**. There are many interesting papers on FAA that lack followup; for example, a study indicating that the cerebellum harbors a FEO (100), interaction between SCN and the dorsomedial hypothalamus using dual lesions in rats (101), the complete lack of FAA in *Nr1d1* neuronal KO mice (56), and the dramatically decreased FAA in melanocortin 3 receptor KO (98), which was not replicated in a separate study by Ribeiro et al. (94). In our own experience, the deficit in FAA displayed by D1R KO mice diminished over time (66, 102). Follow-up studies using modern neurogenetic techniques and/or more refined deletion techniques, using viral vectors in combination with single or dual recombinase systems, could yield greater insights into the FEO using these initial observations as inroads should they prove reproducible. Furthermore, unclear or conflicting results from broad lesion experiments could be further refined with more detailed analyses of these brain regions. For example, c-Fos staining in the supraoptic nucleus and main body of the PVN in the rabbit and rat brain showed activation after feeding, while the posterior PVN showed activation prior to the scheduled feeding time (103, 104). These smaller subregions are more difficult to target and may be treated as a single, larger “region” under less precise techniques.
• **Come to a consensus on a more rigorous definition of “food anticipatory activity”, and be more clear about whether FAA is reduced vs. eliminated**. Could the field agree on a mathematical definition or a heuristic rule for defining FAA? For example, conduct a one-way ANOVA comparing pre- and post-restriction with a significant effect of time? Or a simple rule such as the amplitude of the FAA peak must be at least half that nighttime activity peak to qualify as FAA? (52, 105). Other ideas to consider would be defining the start of FAA to be when activity surpasses the mean, or when an “acceleration” of a certain magnitude occurs (change in activity from previous bin). Given that most researchers collect food entrainment data in different ways, perhaps fitting a logistic curve to data could be useful since there is an upper limit to the amount of FAA that can be expressed. This objective will require a board consensus perhaps assisted by a metaanalysis of FAA or other food entrainment data. Finally, we need to appreciate the difference between a loss vs. a reduction in FAA or other metrics of entrainment. When FAA is reduced, it does not necessarily follow that the timing mechanism, or the FEO, is not working. As we collect more examples of mutations and manipulations that lower FAA, it will be important to measure other aspects of entrainment and potential masking effects (i.e., studies under DD, for example). In the future, it will be important to move beyond activity alone (as measured by running wheels, photobeam breaks, or computer vision) and try to assay motivation to feed using instrumental behavior assays (nose-poking, lever pressing, etc) (43). If we find that all mice with reduced FAA also have reduced instrumental behavior then we can simply use activity, but we cannot make this correlation until we have more examples of animals lacking FAA.

## Author contributions

AS conceived of the manuscript. JT and AS wrote it together. Both authors contributed to the article and approved the submitted version.

## Funding

This work was supported by the National Institute of General Medical Sciences of the National Institutes of Health (SC3GM125570 to AS). The funders had no role in study design, data collection and analysis, decision to publish, or preparation of the manuscript.

## Conflict of interest

The authors declare that the research was conducted in the absence of any commercial or financial relationships that could be construed as a potential conflict of interest.

## Publisher's note

All claims expressed in this article are solely those of the authors and do not necessarily represent those of their affiliated organizations, or those of the publisher, the editors and the reviewers. Any product that may be evaluated in this article, or claim that may be made by its manufacturer, is not guaranteed or endorsed by the publisher.

## Author disclaimer

The content is solely the responsibility of the authors and does not necessarily represent the official views of the National Institutes of Health.
